# Resistance to N-peptide fusion inhibitors correlates with thermodynamic stability of the gp41 six-helix bundle but not HIV entry kinetics

**DOI:** 10.1186/s12977-014-0086-8

**Published:** 2014-10-02

**Authors:** Christopher J De Feo, Wei Wang, Meng-Lun Hsieh, Min Zhuang, Russell Vassell, Carol D Weiss

**Affiliations:** Center for Biologics Evaluation and Research, US Food and Drug Administration, Silver Spring, MD 20993 USA; Present address: Michigan State University, Department of Biochemistry and Molecular Biology, Lansing, MI 48824 USA; Present address: Department of Microbiology, Harbin Medical University, Harbin, Heilongjiang China

**Keywords:** gp41, Fusion, Entry kinetics, Peptide fusion inhibitor, HIV entry, Resistance

## Abstract

**Background:**

The HIV-1 envelope glycoprotein (Env) undergoes conformational changes that mediate fusion between virus and host cell membranes. These changes involve transient exposure of two heptad-repeat domains (HR1 and HR2) in the gp41 subunit and their subsequent self-assembly into a six-helix bundle (6HB) that drives fusion. Env residues and features that influence conformational changes and the rate of virus entry, however, are poorly understood. Peptides corresponding to HR1 and HR2 (N and C peptides, respectively) interrupt formation of the 6HB by binding to the heptad repeats of a fusion-intermediate conformation of Env, making the peptides valuable probes for studying Env conformational changes.

**Results:**

Using a panel of Envs that are resistant to N-peptide fusion inhibitors, we investigated relationships between virus entry kinetics, 6HB stability, and resistance to peptide fusion inhibitors to elucidate how HR1 and HR2 mutations affect Env conformational changes and virus entry. We found that gp41 resistance mutations increased 6HB stability without increasing entry kinetics. Similarly, we show that increased 6HB thermodynamic stability does not correlate with increased entry kinetics. Thus, N-peptide fusion inhibitors do not necessarily select for Envs with faster entry kinetics, nor does faster entry kinetics predict decreased potency of peptide fusion inhibitors.

**Conclusions:**

These findings provide new insights into the relationship between 6HB stability and viral entry kinetics and mechanisms of resistance to inhibitors targeting fusion-intermediate conformations of Env. These studies further highlight how residues in HR1 and HR2 can influence virus entry by altering stability of the 6HB and possibly other conformations of Env that affect rate-limiting steps in HIV entry.

**Electronic supplementary material:**

The online version of this article (doi:10.1186/s12977-014-0086-8) contains supplementary material, which is available to authorized users.

## Background

The HIV-1 envelope protein (Env) mediates virus entry into cells in a multi-step process, presenting many opportunities for blocking HIV infection [1-3]. The gp120 surface subunit of Env initiates the fusion process by interacting with CD4 and chemokine receptors. Subsequent conformational changes allow the transmembrane subunit (gp41) of Env to insert its hydrophobic N-terminus into the target membrane, forming the pre-hairpin intermediate. Self-assembly of the gp41 heptad-repeat regions (HR1 and HR2) then form a thermostable six-helix bundle (6HB). This energetically favorable step facilitates merger of target and virus membranes and stabilizes a fusion pore that enlarges to allow the viral capsid to pass into the target cell cytoplasm [2]. Neutralizing antibodies and fusion inhibitors can interfere with this process, but HIV rapidly mutates and evolves over the course of infection and during treatment to evade these inhibitors [4,5]. The capacity of HIV to mutate hinders efforts to develop broadly effective vaccines and entry inhibitors. Furthermore, escape mutations may alter the functional attributes of Env.

The first entry inhibitor licensed for clinical use, Enfuvirtide (also referred to as T20), is a peptide that mimics the HR2 segment of gp41 (C peptide) [6]. T20 and other similar C peptides bind to the HR1 region of the pre-hairpin intermediate, interrupting 6HB formation in a dominant-negative manner to inhibit fusion [7-16]. However, T20 has a low genetic barrier to resistance, and consequently, resistance develops quickly in patients [6,17-23]. While more potent, new generation C peptides are being developed, they are still susceptible to resistance *in vitro* [24-33]. The common mechanism for escape from C peptides involves mutations within HR1 that destabilize binding of the C peptide to a hydrophobic groove of the HR1 trimeric, coiled-coil core of the 6HB [23,34-39]. Although these mutations necessarily diminish the stability of the 6HB, additional mutations in HR2 can compensate for the fitness cost, and in some cases, can enhance resistance [23,40-43].

Peptides that mimic HR1 (N peptides) are also potent inhibitors, but they are generally less soluble and not yet in clinical use. Their inhibitory mechanism remains unclear, but current models suggest that N peptides can interfere with HR1 coiled-coil formation, and, especially if stabilized as a trimer, can sequester the HR2 region of the pre-hairpin intermediate [44-46]. In either case, as with C peptides, formation of the 6HB is interrupted. HIV can also develop resistance to N peptides, but unlike C peptides, the resistance mutations stabilize the 6HB [46-49]. This finding presents a conundrum because some resistance mutations that increase 6HB stability might also increase peptide inhibitor affinity for gp41 and therefore enhance peptide potency.

However, N-peptide resistance mutations that increase 6HB stability might also increase the rate of 6HB formation relative to peptide inhibition. Indeed, Envs with faster entry kinetics have been reported to be less sensitive to peptide fusion inhibitors [50-52]. Many have attributed this finding to a shorter window of opportunity for peptide accessibility to the pre-hairpin intermediate [50-52]. However, C-peptide fusion inhibitors have thus far not been reported to select for Envs that have faster entry kinetics. Rather, some T20-resistant Envs tended to have overall slower entry kinetics, and only after additional compensatory mutations did entry kinetics reach wild-type levels [40,53]. Irrespective of the resistance mechanism of C-peptides, N peptides select for different resistance mutations, and their effect on Env function is unclear.

In this study, we investigated relationships between virus entry kinetics, 6HB stability, and resistance to peptide fusion inhibitors to gain insights into how residues in HR1 and HR2 can affect Env conformational changes and virus entry. Among the sixteen independent resistant cultures previously selected with one of three different N-peptide inhibitors, two resistance pathways emerged that were defined by having either a glutamic acid to lysine substitution at residue 560 (E560K, HXB2 numbering) in HR1 or a glutamic acid to lysine substitution at residue 648 (E648K, HXB2 numbering) in HR2 [46,48]. Using pseudovirus infectivity and entry assays, we now report that increased 6HB stability, but not faster entry kinetics, correlates with resistance. We also show that increasing 6HB stability is not sufficient to increase the rate of entry. Thus, N-peptide fusion inhibitors do not necessarily select for Envs with faster entry kinetics, nor does faster entry kinetics predict decreased potency of peptide fusion inhibitors. These studies highlight an important role for HR1 and HR2 residues in influencing the relationship between stability of the final fusion-active conformation and other conformations of Env that regulates the rate of virus entry into cells.

## Results

### Effect of different combinations of resistance mutations on Env function

We previously generated escape-mutant viruses selected with peptides corresponding to either 44 (N44) or 36 residues (N36 or the trimer-stabilized IZN36 [54]) in gp41 HR1 and identified two genetic resistance pathways, each defined by a key mutation in either HR1 (E560K) or HR2 (E648K) [46,48]. Each pathway was frequently associated with additional mutations in either the CD4 binding site (E560K pathway) or the V3 loop of gp120 (E648K pathway). To determine whether there were functional relationships between these gp120 and gp41 mutations, we made several chimeric Envs and Envs with site-directed mutations (Table 1). In one set of chimeras, we paired gp41 resistance mutations from one pathway with gp120 mutations from the other pathway. In addition, we made dual-pathway Envs containing the most common HR1 mutations (E560K and Q577R) and the HR2 mutation E648K (WT-KKR, C2-KKR, and C4-KKR) because these pathway-defining mutations were never isolated together despite their high frequency of selection. Finally, we also made single-site mutations that either added or removed C-terminal cytoplasmic tail (C-tail) mutations seen in cultures 4 and 5 to see whether those mutations contribute to the functional properties of Env.

**Table 1:** **Resistance mutations in Env constructs**

**Env category**	**Pathway**	**Construct name** ^***a***^	**gp120 mutations**	**gp41 mutations**		
				**HR1**	**HR2**	**other** ^***b***^
Selected	E560K	C1-C1	L125F, I165K	N554K, E560K, V580L	T641I	
		C2-C2	L125F, E429K	E560K, Q577R		
		C6-C6	V169G	E560K, Q577R	T641I	
	E648K	C3-C3	T319I		E648K	
		C4-C4	T319I, E322D, S306G	Q577R	T641I, E648K	D620N, V833I
		C5-C5	A221V, I309M, D167N		E648K	P714L
Chimeric	E560K	WT-C1		N554K, E560K, V580L	T641I	
		C4-C1	T319I, E322D, S306G	N554K, E560K, V580L	T641I	
		WT-C2		E560K, Q577R		
		C3-C2	T319I	E560K, Q577R		
		WT-C6		E560K, Q577R	T641I	
		E560K			E560K	
	E648K	WT-C3			E648K	
		C2-C3	L125F, E429K		E648K	
		WT-C4		Q577R	T641I, E648K	D620N, V833I
		C1-C4	L125F, I165K	Q577R	T641I, E648K	D620N, V833I
Dual		KKR		E560K, Q577R	E648K	
		C2-KKR	L125F, E429K	E560K, Q577R	E648K	
		C4-KKR	T319I, E322D, S306G	E560K, Q577R	E648K	
C-Tail mutations		C4-C4-T	T319I, E322D, S306G	Q577R	T641I, E648K	D620N
		WT-V833I				V833I
		C5-C5-T	A221V, I309M, D167N		E648K	P714L
		WT-P714L				P714L
WT gp41 constructs		C1-WT	L125F, I165K			
		C2-WT	L125F, E429K			
		C3-WT	T319I			
		C4-WT	T319I, E322D, S306G			

^*a*^Construct named by donor gp120-donor gp41. For example, C1-C4 represents the gp120 from culture C1 and the gp41 from culture C4.

^*b*^Mutations outside of 6HB region.

**Figure 1:**
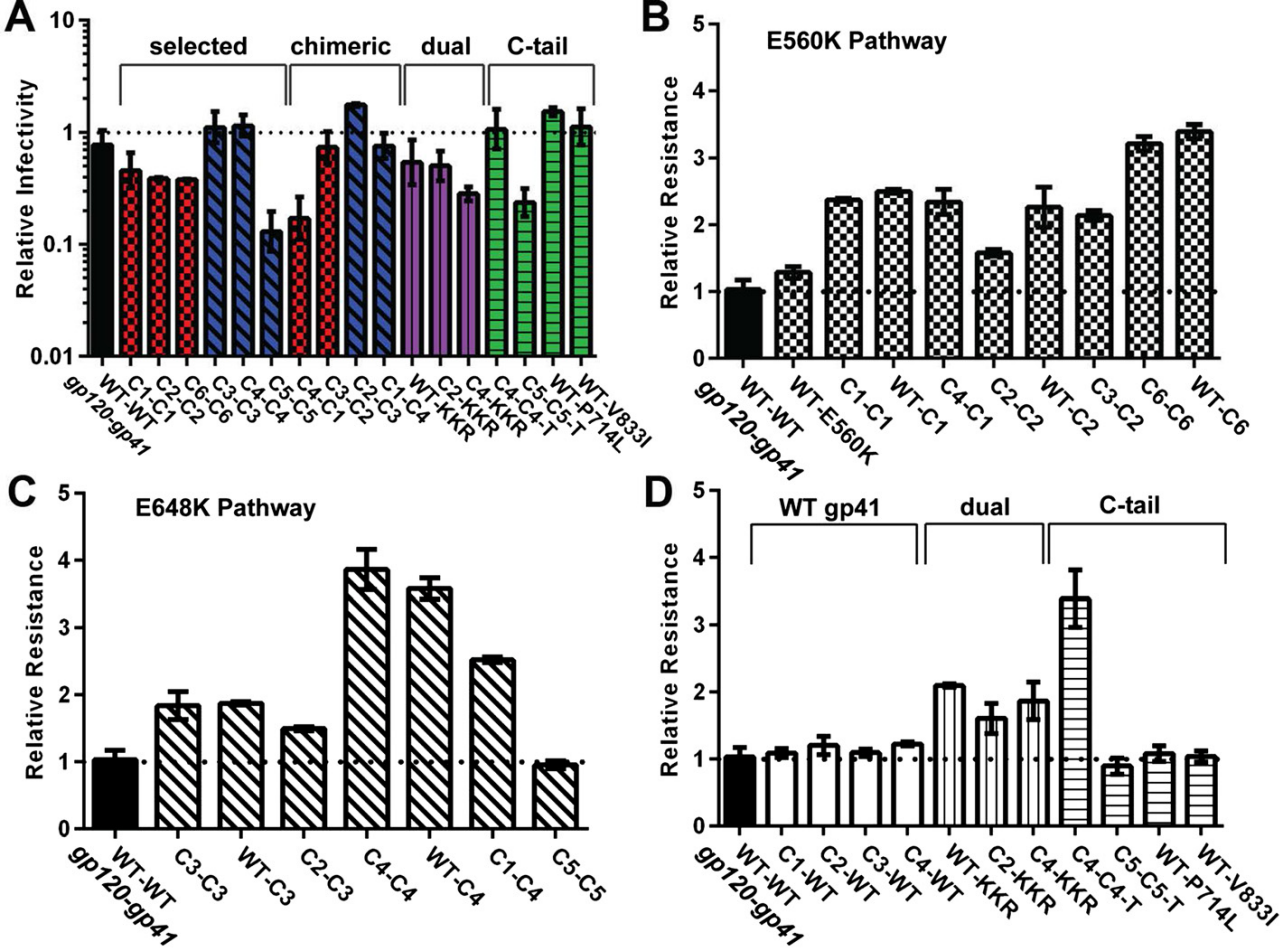
**Chimeric Env pseudotypes containing HR1 and HR2 mutations are functional and confer resistance to N44. (A)** Relative pseudovirus infectivity titered on U87-CD4-CCR5 cells in the absence of selection. Constructs are named by donor gp120-donor gp41. For example, C1-C4 represents the gp120 from C1 and the gp41 from C4. Relative N44 resistance of pseudoviruses bearing Envs with **(B)** E560K pathway mutations (checkered bars), **(C)** E648K pathway mutations (diagonally-hatched bars), or **(D)** Envs with gp120 mutations only (open bars), with both pathway (dual) mutations (vertically-hatched bars), or with mutations to the C-terminal tail (horizontal-hatched bars). Results are normalized to wild type (WT-WT) and averaged across two independent experiments using at least two different pseudovirus lots (±S.E.M). A full description of Env constructs are presented in Table 1.

We first assessed Env function in the pseudotype infectivity assay. Notably, all chimeric Envs (C4-C1, C3-C2, C2-C3, C1-C4), including those Envs with gp41 mutations from both (dual) pathways (WT-KKR, C2-KKR, and C4-KKR), were highly functional and exhibited only modest differences compared with their respective parental (selected) Envs (Figure 1A; Table 2). Reversion of the C-tail mutations (C4-C4-T and C5-C5-T) and the C-tail mutations only (WT-V833I and WT-P714L) also had little effect on infectivity levels. Overall, Envs containing E560K, including both the chimeric Envs (C4-C1, C3-C2) and the selected Envs derived from resistance cultures C1 and C2 (C1-C1, C2-C2, C6-C6), generally conferred lower infectivity than WT Env. In four of five pseudotypes with Envs containing E648K, including the chimeric Envs (C2-C3 and C1-C4) and selected Envs derived from the resistance cultures C3 and C4 (C3-C3 and C4-C4), infectivity was more similar to WT. The exception was the pseudotype with the Env from culture 5 (C5-C5), which had reduced infectivity.

**Table 2:** **Env properties on high CD4 cells**

**Resistance pathway**	**Construct**	**Infectivity**		**Kinetics**		**N44 resistance**	
		**Relative infectivity** ^**a,b**^	***SEM (+/−)***	**T1/2 (min)** ^**c**^	***SEM (+/−)***	**Relative N44 resistance** ^**a,b**^	***SEM (+/−)***
WT	WT-WT	1.00	*0.28*	53.13	*0.61*	1.04	*0.14*
	C1-WT	1.77	*0.11*	61.83	*0.80*	1.09	*0.07*
	C2-WT	1.69	*0.08*	61.39	*2.10*	1.20	*0.13*
	C3-WT	1.07	*0.10*	59.00	*1.26*	1.10	*0.05*
	C4-WT	2.21	*0.11*	58.91	*1.17*	1.23	*0.03*
	WT-V833I^d^	1.28	*0.44*	56.48	*1.80*	1.04	*0.08*
	WT-P714L^d^	1.54	*0.14*	56.68	*3.77*	1.09	*0.12*
E560K	WT-E560K	0.40	*0.04*	58.06	*1.59*	1.29	*0.09*
	WT-C1	0.18	*0.07*	64.98	*0.80*	2.50	*0.03*
	C1-C1	0.51	*0.15*	71.13	*0.64*	2.37	*0.03*
	C4-C1	0.22	*0.08*	69.36	*0.72*	2.34	*0.19*
	WT-C2	0.73	*0.20*	66.98	*2.50*	2.26	*0.30*
	C2-C2	0.39	*0.01*	72.00	*0.41*	1.58	*0.05*
	C3-C2	0.83	*0.30*	66.69	*3.12*	2.14	*0.07*
	WT-C6	0.43	*0.06*	67.36	*1.90*	3.40	*0.11*
	C6-C6	0.38	*0.00*	65.54	*2.19*	3.22	*0.11*
Dual	C2-KKR	0.53	*0.16*	68.54	*2.28*	1.61	*0.23*
	C4-KKR	0.29	*0.04*	67.66	*0.85*	1.87	*0.28*
	WT-KKR	0.60	*0.26*	64.92	*1.50*	2.10	*0.03*
E648K	WT-C3	1.40	*0.14*	48.82	*0.60*	1.88	*0.02*
	C3-C3	1.23	*0.36*	54.41	*1.85*	1.84	*0.21*
	C2-C3	1.75	*0.05*	64.62	*3.90*	1.50	*0.03*
	WT-C4	2.35	*0.65*	52.92	*1.35*	3.59	*0.16*
	C4-C4	1.27	*0.29*	58.06	*1.34*	3.86	*0.30*
	C1-C4	0.81	*0.20*	65.18	*1.76*	2.52	*0.04*
	C4-C4-T^e^	1.23	*0.39*	61.33	*1.91*	3.39	*0.42*
	C5-C5	0.15	*0.05*	69.73	*4.70*	0.96	*0.06*
	C5-C5-T^e^	0.26	*0.06*	70.28	*2.30*	0.90	*0nnn12*

^a^Performed on U87-CD4-CCR5 cells.

^b^Normalization was performed against a different WT pseudovirus lot for each independent experiment.

^c^Performed on JC53 cells.

^d^Mutation in C-terminal tail.

^e^T indicates that the C-terminal tail mutation was removed.

We next tested the chimeric Envs for susceptibility to inhibition by the N44 N-peptide and extended the analysis to include chimeras containing wild-type gp120 or gp41 sequences (Figure 1B-D). Most chimeras displayed resistance levels similar to their respective parental Envs, including the Envs with dual mutations from both pathways. These findings confirm and extend our prior work showing that the gp41 mutations were responsible for resistance. In two cases (C3-C2 compared to C2-C2 and C1-C4 compared to C4-C4 and WT-C4), however, there were modest differences in resistance between the chimera and parental Envs, suggesting that cross-talk between gp120 and gp41 may modulate resistance levels (Figure 1B-C; Table 2).

### Relationship between gp41 6HB stability and resistance

**Figure 2:**
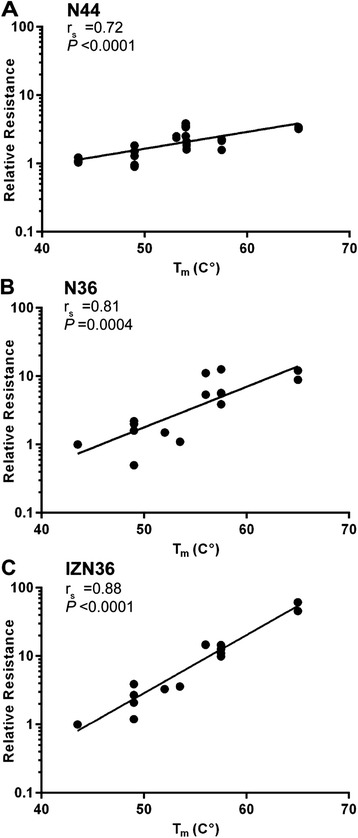
**6HB thermostablity increases with N-peptide resistance.** Scatter plots show correlation between transition mid-point temperature (T*m*) and peptide resistance of pseudoviruses relative to wild type (WT) for **(A)** N44, **(B)** N36, and **(C)** IZN36. Each dot in **(A)** represents the mean resistance for each Env depicted in Figure 1B and C. Results in **(B-C)** were derived from Envs reported previously. Linear regressions and statistics for correlation are indicated for each panel. r_s_, Spearman correlation coefficient.

Using this extended panel of resistant Envs, we next evaluated whether changes in 6HB stability among both selected and chimeric Envs contributed to resistance. We estimated the relative stability of different 6HBs by determining their transition mid-point temperature (T_*m*_) in thermal denaturation studies using mixtures of N and C peptides to model 6HB interactions. Using our previous T_*m*_ values [46,48], as well as additional data obtained in the current study (Table 3), we found a strong correlation between increased resistance and increased 6HB stability (Figure 2A). For example, when the T_*m*_ increased 20°C, resistance increased five-fold. Additionally, using 50% inhibition concentrations and T_*m*_ data from our previous study [46], we found an even stronger relationship between increased 6HB stability and resistance to the other HR1 peptides N36 and IZN36 (Figure 2B and C). Indeed, for IZN36, T_*m*_ increases of 20°C led to a nearly 100-fold increase in resistance to IZN36.

**Table 3:** **Thermal denaturation of N and C peptide mixtures modeling the 6HB**

**Env construct**	**T**_**m**_ **(°C)** ^***a***^
WT-WT	43.5
C1-C1	53.1
C2-C2	57.5
C3-C3	49.0
C4-C4	54.0
C5-C5	49.0
C6-C6	65.0
WT-E560K	49.0
WT-KKR	54.1

^*a*^all but C1-C1 and WT-KKR were determined previously.

### Effect of resistance mutations on entry kinetics

**Figure 3:**
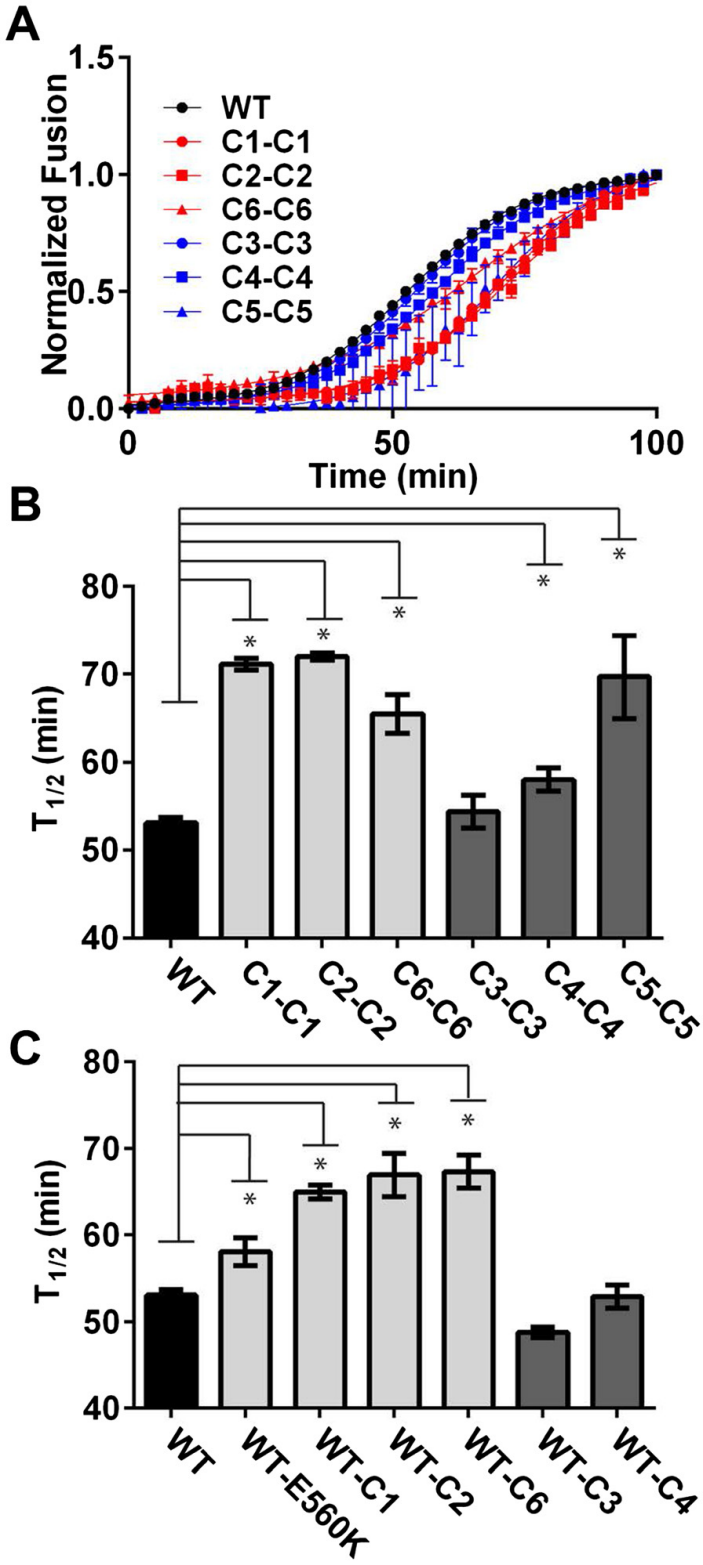
**Pseudoviruses with Envs from resistance cultures have slower entry kinetics than WT. (A)** Entry of β-Lactamase-Vpr pseudoviruses inoculated onto HeLa cells with high CD4 and high CCR5 receptor levels (JC53), normalized to the fusion plateau. Each time point is the average of at least three separate pseudovirus lots tested in independent experiments (±S.E.M). **(B-C)** The time to half maximal (T_1/2_) fusion in target cells comparing **(B)** parental resistant Envs to WT, or **(C)** Env containing only gp41mutations to WT. *unpaired *t*-test indicates *P* values < 0.05.

We next assessed whether increased 6HB stability affected the rate of virus entry, since entry kinetics has been linked to susceptibility to inhibition by peptide fusion inhibitors [50-52]. To measure entry kinetics, we recorded fusion in real time using pseudoviruses that incorporated β-lactamase-Vpr enzyme, which cleaves a fluorescent substrate in the cytoplasm of target cells after virus entry [55-58]. This assay showed that five of six Envs selected in the resistance cultures conferred significantly slower (C1-C1, C2-C2, C6-C6, C4-C4, C5-C5) entry kinetics relative to WT (Figure 3A-B; Table 2). The Env from culture 3 (C3-C3), which had only a single gp41 and gp120 mutation, was more similar to WT. Envs containing HR1 pathway mutations generally showed the slowest entry kinetics, with an entry time to half maximal fusion (T_1/2_) that was ~20-30% slower than WT. The same pattern was seen for Envs containing only the gp41 mutations (Figure 3B-C).

**Figure 4:**
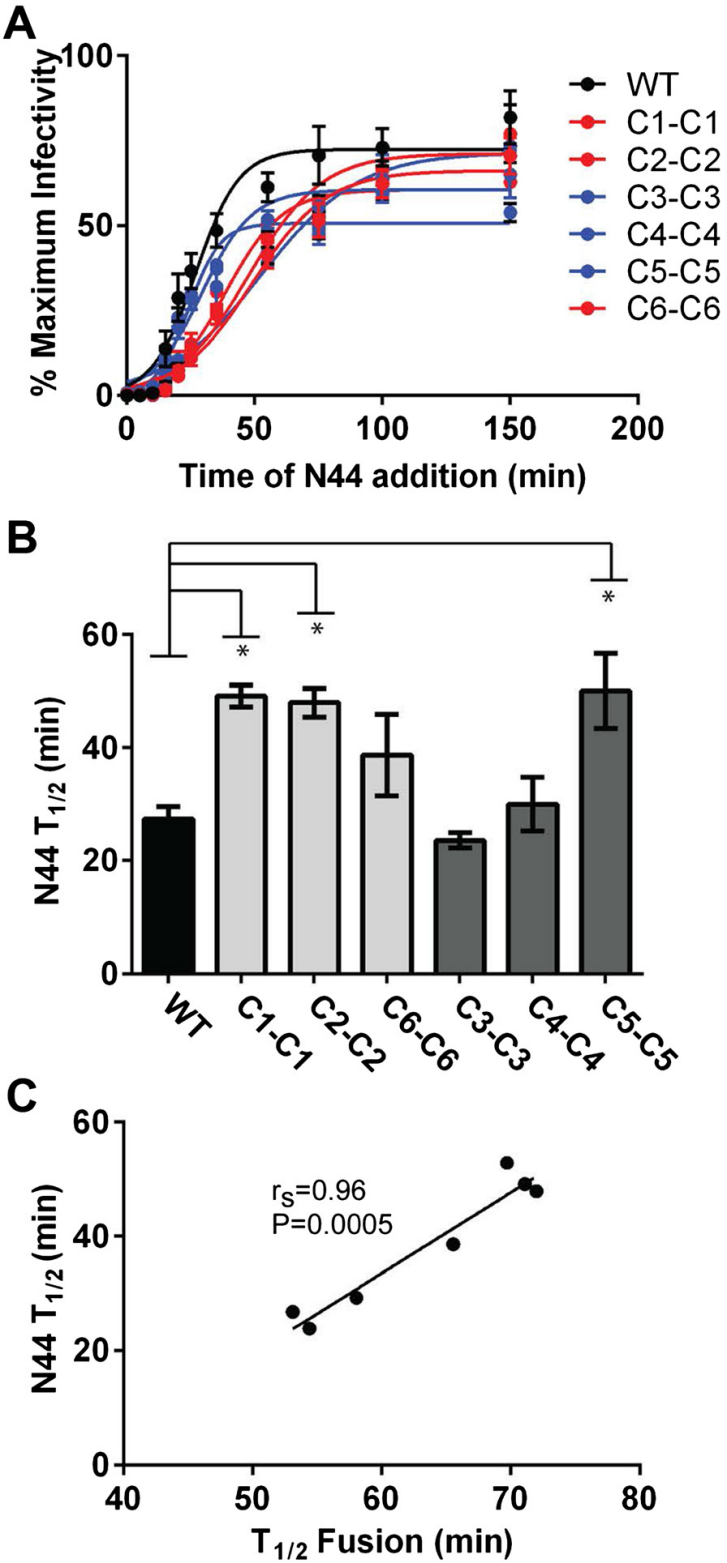
**Resistant Envs do not lose susceptibility to inhibition by N44 faster than WT. (A)** Percent infectivity of pseudovirus on U87-CD4-CCR5 cells after addition of N44 at different time points. **(B)** Calculated T_1/2_ of N44 susceptibility from time of addition experiments in **(A)**. **(C)** Scatter plot correlating the T_1/2_ of N44 susceptibility and the T_1/2_ of fusion (Figure 3). *unpaired *t*-test indicates *P* values <0.05 showing longer time of susceptibility compared to WT. Linear regression and statistics for correlation is indicated. r_s_, Spearman correlation coefficient.

The kinetics results were confirmed in a second assay involving the addition of peptide to cultures at different time points to measure the time during entry when Env is susceptible to peptide inhibition. The peptide prevents fusion after Env binds to CD4 to expose the peptide-sensitive, pre-hairpin, fusion-intermediate conformation of Env and before formation of the 6HB. Therefore, the longer Env remains sensitive to the peptide, the slower it forms the 6HB and fusion pores that drive fusion. Results of these studies showed that pseudotypes with Envs from resistant viruses were not sensitive to inhibition by peptide for shorter periods of time (Figure 4A-B). In particular, pseudotypes with Envs from the E560K pathway (C1-C1, C2-C2) were sensitive to N44 significantly longer than pseudotypes with WT Env (T_1/2_ ~ 75% longer), while the T_1/2_’s of N44 susceptibility for pseudotypes with Envs from the 648 K pathway (C3-C3, and C4-C4) were similar to WT. Thus, both assays indicate that the mechanism of resistance does not require faster entry into cells, and the two assays were strongly correlated with each other (Figure 4C).

### Relationship between entry kinetics and resistance

**Figure 5:**
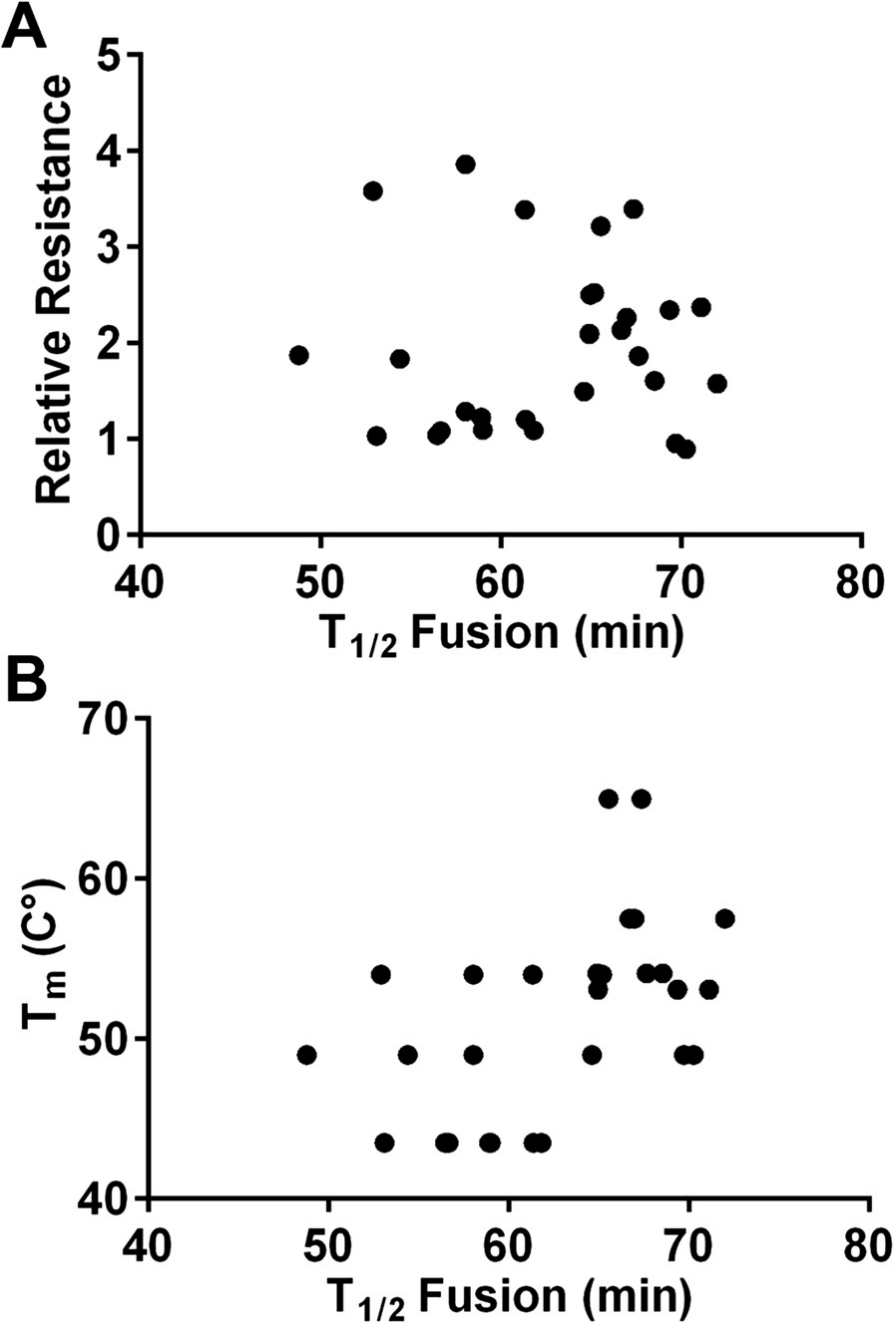
**Pseudovirus entry kinetics does not correlate with resistance or thermodynamic stability of the 6HB. (A)** Scatter plots of the T_1/2_ of fusion and resistance for each pseudovirus described in Table 1. **(B)** Scatter plots of the T_1/2_ fusion value and T_*m*_ of each Env.

Despite prior reports about associations between faster entry kinetics and decreased sensitivity to peptide fusion inhibitors [50-52], we did not find that overall entry kinetics correlated with resistance to the N44 peptide (Figure 5A). Rather, pseudotyped Envs that fused the slowest were still two to three-fold more resistant than WT Envs (Figure 1B and C). 6HB stability and entry kinetics were also not correlated (Figure 5B). This finding was initially surprising because 6HB formation is a major driving force in the fusion process. Nevertheless, this result is consistent with our data showing that N-peptide resistance is correlated with 6HB stability (Figure 2) but not entry kinetics (Figure 5A). Clearly, other Env interactions are more important for controlling the rate-limiting step for entry than the energy contributed by 6HB formation. Mutations that increase bundle stability and confer resistance to N peptides do not necessarily confer inherently faster entry kinetics.

## Discussion

In this study we analyzed relationships between N-peptide resistance, 6HB stability, and entry kinetics to gain insights into how HR1 and HR2 residues affect conformational changes and provide new information on the Env entry and peptide resistance mechanisms. Among our large panel of resistant Envs, we found a correlation between increased 6HB stability and increased resistance, but not between N44 resistance and entry kinetics or time to escape from N44 inhibition. Furthermore, while providing the energy to drive fusion and escape from peptide, the stability of the 6HB was not sufficient to increase the rate of entry kinetics.

Our current study with a large panel of Envs extends our previous work to establish the link between bundle stability and peptide resistance. Previously, we and others have showed that N-peptide resistance mutations that stabilize the 6HB can confer cross-resistance to C peptides (T20 and C34), indicating that N-peptide resistance mutations provide a more universal mechanism of resistance to fusion inhibitors compared to the C-peptide resistance mutations [46,48,49]. Escape mutations that develop after C-peptide selection generally destabilize interactions between Env and C peptides. Perhaps such a direct mechanism of escape is not selected by N peptides because mutations that would disrupt N peptide binding to Env could disrupt both the conserved inner core of HR1 helices, as well as interactions between HR2 and HR1 in the endogenous 6HB needed for virus entry. N peptides have the potential to bind both HR1 and HR2 in the fusion intermediate, so more than one mutation may be needed to directly disrupt N peptide binding to Env [59]. Therefore, the more universal mechanism of improving the stability of the endogenous 6HB bundle to favor the virus might be a more efficient resistance mechanism, especially for an inhibitor that can bind to two different sites of Env.

By contrast, C peptides may not select for such an escape mechanism because resistance mutations in the exposed surface of the HR1 coiled coil that confer high-level resistance to C peptides may not be overly detrimental to 6HB formation. Nonetheless, secondary C-peptide resistance mutations in HR2 do increase 6HB stability and may directly contribute to resistance, but these mutations generally occur after the primary resistance mutations [23,40-43]. Indeed, a more potent C peptide inhibitor with increased affinity for the 6HB selects a more complex profile of resistance mutations, suggesting that resistance mutations that are needed to directly compete for the higher affinity binding of the inhibitor may impose a fitness cost on Env function [26]. These examples highlight the complex relationship between mutations that directly confer resistance and mutations that affect the inherent kinetics and efficiency of Env entry. The interplay between these attributes determines the overall fitness of the resistant virus.

Notably, increasing 6HB stability confers resistance without increasing the rate of virus entry. This finding was initially unexpected because Envs that confer faster entry have been reported to be less sensitive to peptide fusion inhibitors than Envs that confer slower entry [50-52]. In addition, increased stability of the 6HB should facilitate formation of the final fusion-active conformation of Env. Two considerations may help to address the issue of why increasing 6HB stability doesn’t increase entry kinetics. First, high 6HB stability may increase the likelihood of premature 6HB formation and inactivation of Env, resulting in fewer functional Env trimers. This could slow entry kinetics if several functional Env trimers need to coalesce to form a fusion pore. Second, most kinetic experiments, including our own, report overall entry kinetics rather than the half-life of discreet, peptide-sensitive fusion intermediates. In fact, the rate constant for early steps in the fusion process, and the relative competition between peptide association rates and intermediate lifetimes, are both important for defining peptide sensitivity [60,61]. It therefore remains possible that the 6HB-stabilizing mutations shorten the lifetime of peptide-sensitive conformational intermediates without speeding up the overall fusion process.

Our experiments measuring time of escape from N44 inhibition also indicate that resistant Envs do not lose susceptibility to N44 inhibition faster than WT Env. Two alternate explanations might account for this observation. In one case, the peptide-sensitive, pre-hairpin intermediate might indeed have a longer lifetime, but resistance can be explained by an equilibrium between the peptide-sensitive, pre-hairpin intermediate and a later, pre-bundle conformation that is insensitive to peptide. Increased bundle stability would favor the later peptide-insensitive conformation and thus fusion in the presence of peptide. Alternatively, increasing bundle stability could increase the rate of 6HB formation and shorten the lifetime of the sensitive intermediate, but the bundle-stabilizing mutations could additionally affect a different conformation of Env, resulting in slower entry kinetics. For example, the resistance mutations might also impair receptor interactions or subsequent conformational changes and delay the start of the window of susceptibility to peptide, without changing the length of time of the window of susceptibility. The recent pre-fusion structure of trimeric Env supports such a possibility because gp41, and especially HR1, makes intimate contact with the inner domain of gp120 [62-65]. The time of escape from peptide inhibition experiments therefore could reflect the rate-limiting steps prior to peptide inhibition and make the window of peptide susceptibility look longer in that it ends later, even if the length of the window is actually unchanged or shorter.

## Conclusions

In summary, we show that mutations in HR1 and HR2 that confer resistance to peptide fusion inhibitors increase 6HB stability without conferring increased entry kinetics. Our data indicate that increased 6HB stability does not necessarily enhance entry kinetics and that N peptide inhibitors do not select for Envs with faster entry kinetics. These studies highlight an important role for HR1 and HR2 residues in influencing the relationship between stability of the final fusion-active conformation and other conformations of Env that regulates the rate of virus entry into cells.

## Methods

### Cells

293 T cells and U87 cells expressing high levels of CD4 and CCR5 (U87-CD4-CCR5) were provided by Dan Littman (New York University, New York, NY). HeLa cells expressing high levels of CD4 and CCR5 (JC53) were provided by David Kabat (Oregon Health and Science University, Portland, OR). 293 T and HeLa cells were passaged in Dulbecco’s modified Eagle’s medium (DMEM; Mediatech, Inc, Manassas, VA) supplemented with 10% fetal bovine serum (FBS, Atlanta Biologicals, Lawrenceville, GA), 1× penicillin, 1× streptomycin, 2 mM glutamine, 1 mM HEPES buffer, and 1× non-essential amino acids (DMEM^+^). U87-CD4-CCR5 cells were passaged in DMEM^+^ supplemented with 300 μg/ml G418 Sulfate (Mediatech, Inc, Manassas, VA) and 1 μg/ml puromycin (Sigma, St. Louis, MO).

### Plasmids/constructs

Env-deficient HIV-1 genome (gag/pol) plasmid pCMVΔR8.2, luciferase (Luc) reporter plasmid pHR’CMV-Luc, and expression vector pCMV/R were obtained from Gary J. Nabel (NIH, Bethesda, MD) and described previously [48]. pMM310-β-lactamase-Vpr reporter plasmid was obtained from Gregory B. Melikyan (Emory Children's Center, Atlanta, GA). JR-CSF wild-type (WT) and resistant Envs were cloned into the pCMV/R vector as previously described [46,48]. Chimeras between the gp120 and gp41 of select Envs were constructed by swapping segments using Mfe1 and Not1 restriction sites within the construct. C-terminal tail mutations were incorporated using standard site-direct mutagenesis techniques. The entire *env* gene was sequenced to confirm that the desired mutations were made.

### Pseudovirus production

293 T cells were seeded at 3.25 × 10^5^cells/mL in a 10-cm dish approximately 20 h prior to transfection. Pseudovirus stocks were obtained by transfecting cells with 0.5 μg pCMV/R-Env, and either 4 μg each of pCMVΔR8.2 and pHR’CMV-Luc, or 10 μg of pCMVΔR8.2 and 5 μg of pMM310-b-lactamase-Vpr, together with Fugene 6 according to the manufacturer’s recommendations (Promega, Madison, WI). Media was changed at 18–20 h and at 48 h post-transfection, supernatants were harvested and filtered through a 0.45-μm pore-size low protein binding filter. Virus stocks were quantified by HIV-1 p24 Gag ELISA (AIDS Vaccine Program, NCI-Frederick Cancer Research and Development Center, Frederick, MD) and stored at −80°C.

### Infectivity assay

U87-CD4-CCR5 cells were plated at 2 × 10^4^ cells per well in white-walled, 96-well plates (Nunclon Delta Surface, Thermo Scientific, Denmark) and spun at 200 × *g* in a Sorvall RT 6000D centrifuge (Dupont, Wilmington, DE) for 3 min at 25°C the day prior to infection. Seven steps of two-fold serial dilutions of pseudoviruses containing 8 μg/ml polybrene (Sigma-Aldrich, St. Louis, MO) were applied to cells for infection. After 24 h, an additional 100 μl of warm DMEM^+^ was added to each well. At ~48 h post-infection, cells were lysed with buffer (Luciferase Cell Culture Lysis 5x reagent, Promega, Madison, WI), and luciferase activity was assayed using kit reagents (Luciferase Assay System, Promega, Madison, WI) according to the manufacturer’s instructions. To determine the infectivity titer, luciferase activity for replicate means of each dilution within the linear range were normalized by p24 Gag content and averaged across dilutions. The infectivity of at least two different pseudovirus lots was determined from separate experiments and normalized to the average WT titer.

### Peptide inhibition assay

The N44 peptide dose–response assay was performed as described above except 300 RLU of pseudovirus was used and peptide was added prior to adding to cells. The N44 peptide was synthesized using standard 9-fluorenylmethoxy carbonyl chemistry and purified by high-pressure liquid chromatography (CBER Facility for Biotechnology Resources, Bethesda, MD). The sequence is based on residues 547 to 590 (HXB2 numbering), except for an isoleucine to glycine change at position 548 (GGVQQQNNLLRAIEAQQHLLQLTVWGIKQLQARILAVERYLKDQ). The 50% inhibitory concentration (IC_50_) of N44 was determined from dose–response curves using non-linear regression analysis in Graph Pad Prism 6 (Graph Pad Prism Software, San Diego, California). Experiments were performed in duplicate and at least two different pseudovirus lots were used in independent experiments to determine the sensitivity to N44. The IC_50_values were normalized against WT for each individual experiment before averaging across experiments to give the mean relative resistance.

### Kinetic assay

JC53 cells, frequently used for beta-lactamase kinetic assays, were plated at 2 × 10^4^ per well in clear-bottom, black-walled, 96-well plates (Costar, Corning Inc., Corning, NY) the day prior to the assay [55-57,61]. Beta-lactamase loading solution (Life Technologies, Madison, WI) was prepared at room temperature using DMEM (minus phenol red) supplemented with 25 mM HEPES, 2 μM CCF2-AM Dye (Solution A), 10 μL per mL of Solution B, 157 μL per mL of Solution C, and 3.1 mM probenecid (Sigma-Aldrich, St. Louis, MO). Cells were washed with Hanks Buffered Saline Solution (HBSS; Mediatech, Inc, Manassas, VA), prior to adding 50 μl of loading substrate to each well. Cells were allowed to take up substrate for 1 h at room temperature in the dark prior to placing on ice. Meanwhile, β-lactamase-containing pseudoviruses were first thawed at room temperature, then placed on ice and diluted into ice-cold DMEM^+^ supplemented with probenecid and polybrene for final concentrations of 2.5 mM and 8 μg per mL, respectively. After this initial 1.3-fold dilution, three additional 1.4-fold serial dilutions of each pseudovirus were applied in duplicate to cells that had been washed twice with HBSS containing 2.5 mM probenecid. These dilutions spanned the entire dynamic range of the assay for most pseudoviruses tested (Additional file 1: Figure S1). All manipulations were performed on ice. The 96-well plate was placed on a thermo-conductive aluminum block covered with a wet paper towel and immersed in ice to keep the cells cold throughout washing and pseudovirus application. Pseudoviruses were spinoculated in the Sorvall RT 6000D centrifuge for 30 min at 4°C and 2500 × *g* and then immediately placed into a Spectra Max Gemini EM (Molecular Devices, Sunnyvale, CA) fluorescence plate reader at 37°C. Florescence changes were monitored over time using a 409 nm excitation and both a 460 nm (blue) and 528 nm (green) emission detection. We measured fusion for 100 minutes to capture maximum fusion, which was comparable to time points reported by others using similar assays [56,58]. We also noticed that control pseudoviruses without Env produced a time-dependent increase in background signal as incubation periods approach 100 min (Additional file 1: Figure S1).

To obtain the working fusion signal in florescence units, the blue:green ratio for wells mock treated only with DMEM^+^ was subtracted from the blue:green ratio of wells treated with pseudovirus. The signal at each time point was normalized to the signal at 100 min. The normalized signal for each replicate was averaged prior to averaging across the four dilutions to give the final entry kinetic curve. This trace was fitted to a 4-parameter logistic equation to obtain the T_1/2_ (min) for each pseudovirus using Graph Pad Prism 6. Three independent measurements of time to half-maximal fusion (T_1/2_) were determined for each Env using at least three different pseudovirus lots.

### Time to escape N44 inhibition

U87-CD4-CCR5 cells were plated as described above for the infectivity assay. The following day the cells were cooled on ice, and media was removed. 100 μl of pseudovirus containing 8 μg/mL of polybrene were spinoculated in the Sorvall RT 6000D centrifuge for 30 min at 4°C and 2500 × g. Pseudovirus was then removed and 75 μl of DMEM^+^ warmed to 37°C was added to start infectivity and placed into a 37°C incubator. 25 μl of 20 μM (excess) N44 was then added to the cells at times corresponding to 0, 5, 10, 15, 20, 25, 35, 55, 75, 100, and 150 min incubations at 37°C. Additionally, 25 μl of diluent without N44 was added at 150 min to serve as a control to normalize for total infectivity. 24 h later, DMEM^+^ warmed to 37°C was added, and the assay plate was allowed to incubate for another 24 hr. Luciferease activity was then assayed as described above and normalized to the infectivity of the 150 min time point without peptide. Samples were run in duplicate, and at least two independent experiments were performed using different pseudovirus lots. The time course was fit to a 4-parameter logistic equation with a constraint that the bottom asymptote must be equal to zero to determine the T_1/2_ (min) using Graph Pad Prism 6. All independent measures of T_1/2_ (min) were then averaged.

### Circular dichroism experiments

6HB stability studies were performed as previously described [46,48]. In this study, 10 μM of each HR1 and HR2 peptide (KKR- and C1-mutant, respectively) were mixed in sodium phosphate buffer (PBS) at room temperature. A Jasco spectropolarimeter (model J-810, Jasco Inc., Easton, MD) was used to collect circular dichroism (CD) spectra of this peptide mixture. Thermal denaturation was monitored at 220 nm between 4° and 95°C. The transition mid-point temperature (T_*m*_) was estimated from the first-derivative of the denaturation curves using Jasco software utilities.

### Statistical analysis

Spearman correlation coefficients were used to determine correlations, and *t*-tests were performed using GraphPad Prism software. *P* values < 0.05 were considered significant. Comparisons were made using mean values from different pseudovirus lots for a particular Env. Unpaired, parametric two-tailed *t*-tests were used as indicated in the figure captions.

## Additional file

Additional file 1: Figure S1. Raw kinetic data. **(A)** Representative real time entry for four serial dilutions (1.3-fold to 3.6-fold) of the WT β-lam pseudovirus. **(B)** Representative real time entry for the lowest dilutions of either WT Env, C1-C1 Env, or the no HIV Env control β-lam pseudoviruses. **(C)** Entry kinetics for WT-WT and C1-C1 using the no HIV Env as the background control. Here the kinetic relationships are preserved using this alternate background correction. Error bars, standard deviation of mean for each time point. Inset, T_1/2_ of each of the curves in (±95% C.I).

## Abbreviations

Env: Envelope protein
gp41: HIV envelope transmembrane subunit
gp120: HIV envelope surface subunit
HR1 and HR2: Heptad-repeat regions one and two
6HB: Thermostable six-helix bundle
T20: Enfuvirtide
C peptide: HR2 segment of gp41
N peptide: HR1 segment of gp41
N44: N Peptide 44 residue long
N36: N peptide 36 residues long
IZN36: Trimer stabilized N peptide
C-tail: C-terminal cytoplasmic tail
T_*m*_: Transition mid-point temperature
T_1/2_: Time to half maximal fusion
