# Supplementary material for: Resistance to N-peptide fusion inhibitors correlates with thermodynamic stability of the gp41 six-helix bundle but not HIV entry kinetics
Source: Retrovirology. 2014 Oct 2;11:86. doi: 10.1186/s12977-014-0086-8 (PMC4190581; doi:10.1186/s12977-014-0086-8)
Supplement: Additional file 1: Figure S1. — Raw kinetic data. (A) Representative real time entry for four serial dilutions (1.3-fold to 3.6-fold) of the WT β-lam pseudovirus. (B) Representative real time entry for the lowest dilutions of either WT Env, C1-C1 Env, or the no HIV Env control β-lam pseudoviruses. (C) Entry kinetics for WT-WT and C1-C1 using the no HIV Env as the background control. Here the kinetic relationships are preserved using this alternate background correction. Error bars, standard deviation of mean for each time point. Inset, T1/2 of each of the curves in (±95% C.I). [file 12977_2014_86_MOESM1_ESM.pdf]

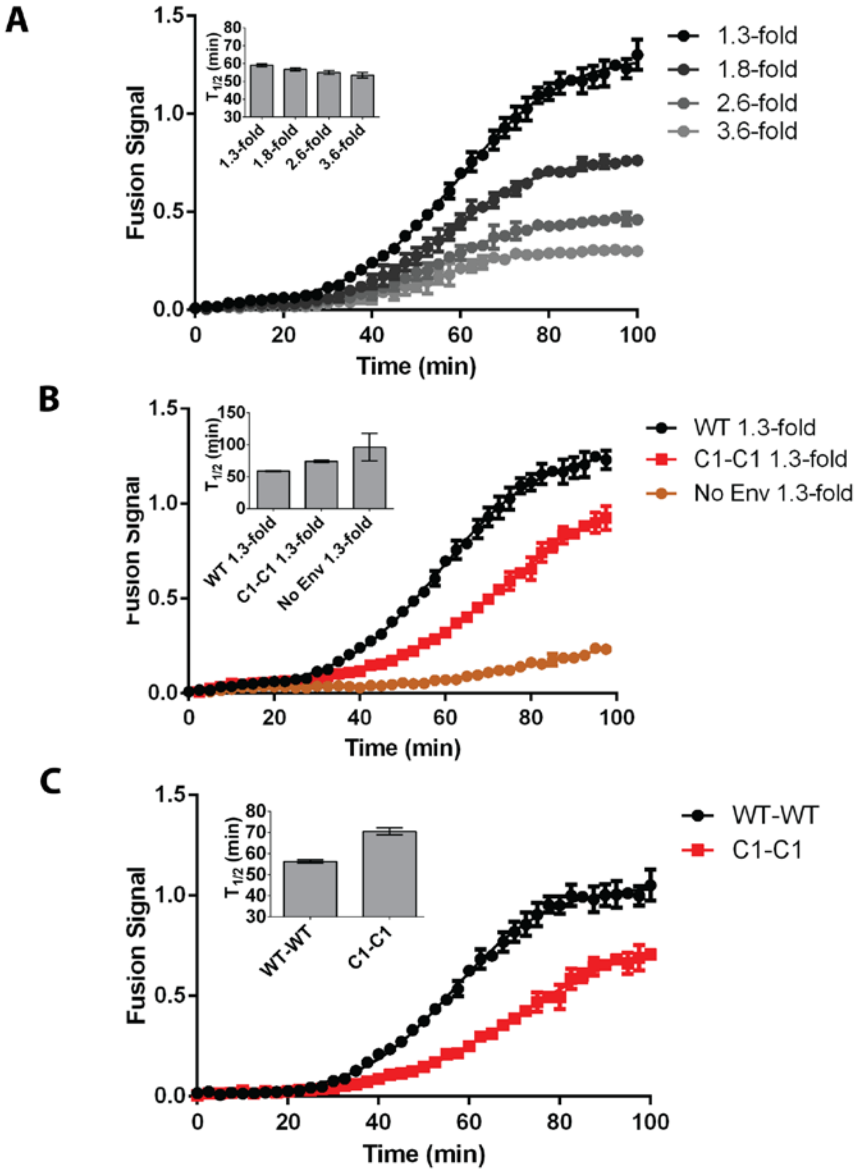

Figure S1 Raw kinetic data. (A) Representative real time entry for four serial dilutions (1.3-fold to 3.6-fold) of the WT β-lam pseudovirus. (B) Representative real time entry for the lowest dilutions of either WT Env, C1-C1 Env, or the no HIV Env control β-lam pseudoviruses. (C) Entry kinetics for WT-WT and C1-C1 using the no HIV Env as the background control. Here the kinetic relationships are preserved using this alternate background correction. Error bars, standard deviation of mean for each time point. Inset, $T_{1/2}$ of each of the curves in (±95%C.I).
